# ALDH2 as a potential stem cell-related biomarker in lung adenocarcinoma: Comprehensive multi-omics analysis

**DOI:** 10.1016/j.csbj.2023.02.045

**Published:** 2023-02-24

**Authors:** Thi-Oanh Tran, Thanh Hoa Vo, Luu Ho Thanh Lam, Nguyen Quoc Khanh Le

**Affiliations:** a International Ph.D. Program for Cell Therapy and Regeneration Medicine, College of Medicine, Taipei Medical University, Taipei 110, Taiwan; b Hematology and Blood Transfusion Center, Bach Mai Hospital, No.78, Giai Phong street, Hanoi, Viet Nam; c Department of Science, School of Science and Computing, South East Technological University, Waterford X91 K0EK, Ireland; d Pharmaceutical and Molecular Biotechnology Research Center (PMBRC), South East Technological University, Waterford X91 K0EK, Ireland; e Department of Pediatrics, Pham Ngoc Thach University of Medicine, Ho Chi Minh city, Viet Nam; f Children’s Hospital 1, Ho Chi Minh city, Viet Nam; g Professional Master Program in Artificial Intelligence in Medicine, College of Medicine, Taipei Medical University, Taipei 106, Taiwan; h Research Center for Artificial Intelligence in Medicine, Taipei Medical University, Taipei 106, Taiwan; i Translational Imaging Research Center, Taipei Medical University Hospital, Taipei 110, Taiwan

**Keywords:** 4-HNE, 4-Hydroxynonenal, AJCC, American Joint Committee On Cancer, ALDH, Aldehyde Dehydrogenase, CGI, Cpg Island, CPTAC, Clinical Proteomic Tumor Analysis Consortium, CSCs, Cancer Stem Cells, IHC, Immunohistochemical, LCSCs, Liver Cancer Stem Cells, LUAD, Lung Adenocarcinoma, MAPK, Mitogen-Activated Protein Kinase, MDA, Malondialdehyde, NSCLC, Non-Small Cell Lung Cancer, OS, Overall Survival, ROS, Reactive Oxygen Species, SCLC, Small Cell Lung Cancer, TCGA, The Cancer Genome Atlas, TMT, Tandem Mass Tags, TNM, Tumor-Node-Metastasis, UICC, International Union For Cancer Control, XRCC1, X-Ray Repair Cross-Complementing Protein 1, Aldehyde Dehydrogenase 2, Cancer stem cells, DNA methylation, Gene expression, Lung adenocarcinoma, Protein expression, Survival analysis

## Abstract

Lung adenocarcinoma (LUAD) is the most prevalent lung cancer and one of the leading causes of death. Previous research found a link between LUAD and Aldehyde Dehydrogenase 2 (ALDH2), a member of aldehyde dehydrogenase gene (ALDH) superfamily. In this study, we identified additional useful prognostic markers for early LUAD identification and targeting LUAD therapy by analyzing the expression level, epigenetic mechanism, and signaling activities of ALDH2 in LUAD patients. The obtained results demonstrated that ALDH2 gene and protein expression significantly downregulated in LUAD patient samples. Furthermore, The American Joint Committee on Cancer (AJCC) reported that diminished ALDH2 expression was closely linked to worse overall survival (OS) in different stages of LUAD. Considerably, ALDH2 showed aberrant DNA methylation status in LUAD cancer. ALDH2 was found to be downregulated in the proteomic expression profile of several cell biology signaling pathways, particularly stem cell-related pathways. Finally, the relationship of ALDH2 activity with stem cell-related factors and immune system were reported. In conclusion, the downregulation of ALDH2, abnormal DNA methylation, and the consequent deficit of stemness signaling pathways are relevant prognostic and therapeutic markers in LUAD.

## Graphical Abstract

**Figure:**
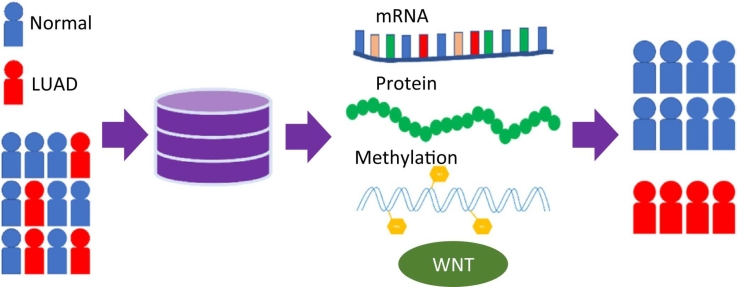

## 1 Background

Lung cancer is a major source of cancer incidence and death globally [1]. This cancer is divided into two types: small cell lung cancer (SCLC), which accounts for 15% of cases, and non-small cell lung cancer (NSCLC), which accounts for 85% of cases. The most common primary lung cancer in the United States is lung adenocarcinoma (LUAD), a subtype of NSCLC [2]. Despite all efforts at multimodal therapy over the past several decades, the survival time of LUAD patients has shown a minor increase. It is worth noting that recurrence and metastasis are common in LUAD [3]. Therefore, it is critical to identify new biomarkers for the early stages of LUAD and novel therapeutic targets for LUAD treatment.

ALDH2 (aldehyde dehydrogenase 2) is an enzyme that belongs to the aldehyde dehydrogenase gene (ALDH) superfamily [4], [5]. The ALDH2 family has only one member, which encodes the mitochondrial ALDH with the highest affinity for acetaldehyde and is essential in ethanol metabolism [6]. Previous research has linked ALDH2 dysfunction and suppression to a variety of human diseases and cancers. For example, Chen et al. (2018) described that ALDH2 expression levels are associated with high X-ray repair cross-complementing protein 1 (XRCC1) expression levels and may serve as a useful prognostic tool in lung and hepatocellular cancer [7]. Also, in 2019, Li et al. reported that ALDH2 repression promotes lung tumor progression via accumulated acetaldehyde and DNA damage [8]. In addition, methylation-induced silencing of ALDH2 can promote LUAD bone metastases by stimulating the mitogen-activated protein kinase (MAPK) pathway [9]. However, despite the importance of ALDH2 in lung cancer, the epigenetic profile and signaling pathways that resulted in ALDH2 abnormal expression in LUAD remain little understood.

In current study, via multi-bioinformatics analyses [10], we aimed to investigate (1) the gene and (2) protein expression of ALDH2 in LUAD patients. Then, we examine (3) the prognosis value of ALDH2 based on different clinicopathological factors. Furthermore, we investigated (4) the DNA methylation status and (5) the signaling pathways involved in ALDH2 activation. Additionally, Toledo-Guzmán et. al (2019) reported the evidences for ALDH family members as a cancer stem cells (CSCs) marker in solid tumors [11]. Hence, we decided to focus on examining (6) the relationship of ALDH2 activity with stem cell-related factors and with the immune cells.

## 2 Materials and methods

### 2.1 Analysis of the interested gene expression

UALCAN was employed to examine gene expression in several forms of cancer. (http://ualcan.path.uab.edu) [12]. Firstly, we investigated the expression of ALDH2 cross 24 different cancer types using data from The Cancer Genome Atlas (TCGA) [13]. Then, with the aim to investigate the ALDH2 level in LUAD patients, TCGA-LUAD dataset that includes 585 cases was used. The more detail about the dataset can be found on TCGA portal with following link https://portal.gdc.cancer.gov/. We provided graphs and plots depicting expression profiles for result visualization.

### 2.2 Protein profile investigation

We also performed UALCAN analysis to study the expression of ALDH2 protein in LUAD tissue and investigate the potential signaling pathways that involved in the activation of ALDH2. CPTAC-LUAD dataset that collected from Clinical Proteomic Tumor Analysis Consortium (CPTAC) was used. The dataset includes 111 cases (with 102 tumors paired with normal adjacent tissue samples) and is available in https://pdc.cancer.gov/pdc/ (study ID: PDC000153). Protein expression of ALDH2 in LUAD tissues were quantified by mass spectrometry of tissues using isobaric tags (TMT (tandem mass tags)− 10) methods, the method protocol is available in [14]. This dataset also was used for investigating ALDH2 expression profile base on different pathways (HIPPO Pathway, mTOR Pathway, MYC/MYCN Pathway, RTK Pathway, SWI-SNF complex, WNT Pathway, p53/Rb-related pathway, and NRF2 Pathway).

To visualize the expression of ALDH2 protein in LUAD tissues, we went to https://www.proteinatlas.org, The Human Protein Atlas (HPA), to examine ALDH2 pathology. This is a valuable resource for studying protein localization and expression in human tissues and cells [15]. We collected immunohistochemical (IHC) images and protein expression profile of six LUAD patients. HPA051065 antibody was used for staining. The protein expression level determined by immunohistochemistry was then classified as strong, moderate, weak, or negative. Patient tissue samples were collected from the Department of Pathology at Uppsala University Hospital in Uppsala, Sweden, as part of the sample collection administered by the Uppsala Biobank (http://www.uppsalabiobank.uu.se/en/). Pathologists examined all pictures in the submitted dataset and interpreted them by staining intensity and percentage of positive cancer cells. Detailed information of patients is listed in Supplementary Table S1.

### 2.3 Overall survival analysis

Kaplan-Meier Plotter (KM Plotter) was used to examine the correlation between interested gene expression and OS of LUAD patients. We explored the connections between ALDH2 expression and clinical prognosis in LUAD based on different clinicopathological variables. The hazard ratio (HR) with 95% CI and log-rank P value were computed.

### 2.4 DNA methylation analysis

To investigate the methylation phenotype of ALDH2 in LUAD samples, we used the SMART software (http://www.bioinfo-zs.com/smartapp). Based on TCGA data, SMART (Shiny Methylation Analysis Resource Tool) provides comprehensive DNA methylation analysis and visualization plots [16]. TCGA-LUAD dataset for DNA methylation analysis includes 30 normal and 458 tumor samples. To give the general information about interested gene, we perform SMART summary to illustrate chromosomal distribution and location of methylation probes. To evaluate epigenetic regulation of ALDH2 across different types of cancer, we performed pan-cancer gene expression analysis, setting parameters: Median; M-value. To compare the DNA methylation probe-level between tumor and normal tissue, we conducted methylation box plot, setting parameters: Median; M-value.

### 2.5 TIMER analysis

TIMER (https://cistrome.shinyapps.io/timer/) was used to investigate the relationship between gene-gene expression and the clinical relevance of ALDH2 to immune cells. We also investigated the relationship between the targeted gene and the immune purity, T cells, B cells, Macrophage, Neutrophil, and dendritic cells.

## 3 Results

ALDH2 gene and protein information is summarized in Table 1. In short, ALDH2 is located on the long arm of human chromosome 12 at the locus 12q24.2. ALDH2 is comprised of 517 amino acids. This protein is an electron carrier in the cellular mitochondrion matrix; however, its specific function is unknown.

**Table 1:** ALDH2 gene and protein information.

Name	Aldehyde dehydrogenase, mitochondrial
Gene Name	ALDH2
Synonyms	1.2.1.3; ALDH class 2; ALDH-E2; ALDHI; ALDM
Organism	Humans
Amino acid sequence	MLRAAARFGPRLGRRLLSAAATQAVPAPNQQPEVFCNQIFINNEWHDAVSRKTFPTVNPSTGEVICQVAEGDKEDVDKAVKAARAAFQLGSPWRRMDASHRGRLLNRLADLIERDRTYLAALETLDNGKPYVISYLVDLDMVLKCLRYYAGWADKYHGKTIPIDGDFFSYTRHEPVGVCGQIIPWNFPLLMQAWKLGPALATGNVVVMKVAEQTPLTALYVANLIKEAGFPPGVVNIVPGFGPTAGAAIASHEDVDKVAFTGSTEIGRVIQVAAGSSNLKRVTLELGGKSPNIIMSDADMDWAVEQAHFALFFNQGQCCCAGSRTFVQEDIYDEFVERSVARAKSRVVGNPFDSKTEQGPQVDETQFKKILGYINTGKQEGAKLLCGGGIAADRGYFIQPTVFGDVQDGMTIAKEEIFGPVMQILKFKTIEEVVGRANNSTYGLAAAVFTKDLDKANYLSQALQAGTVWVNCYDVFGAQSPFGGYKMSGSGRELGEYGLQAYTEVKTVTVKVPQKNS
Number of residues	517
Molecular Weight	56,380.93
Theoretical pI	7.05
Functions	aldehyde dehydrogenase (NAD) activity / aldehyde dehydrogenase [NAD(P)+ ] activity / electron carrier activity
Processes	alcohol metabolic process / carbohydrate metabolic process / ethanol catabolic process / ethanol oxidation / neurotransmitter biosynthetic process / small molecule metabolic process / synaptic transmission / xenobiotic metabolic process
General Function	Electron carrier activity
Specific Function	Not Available
Pfam Domain Function	Aldedh (PF00171)
Cellular Location	Mitochondrion matrix
Chromosome Location	12
Locus	12q24.2

### 3.1 The downregulation of ALDH2 in LUAD samples

**Fig. 1:**
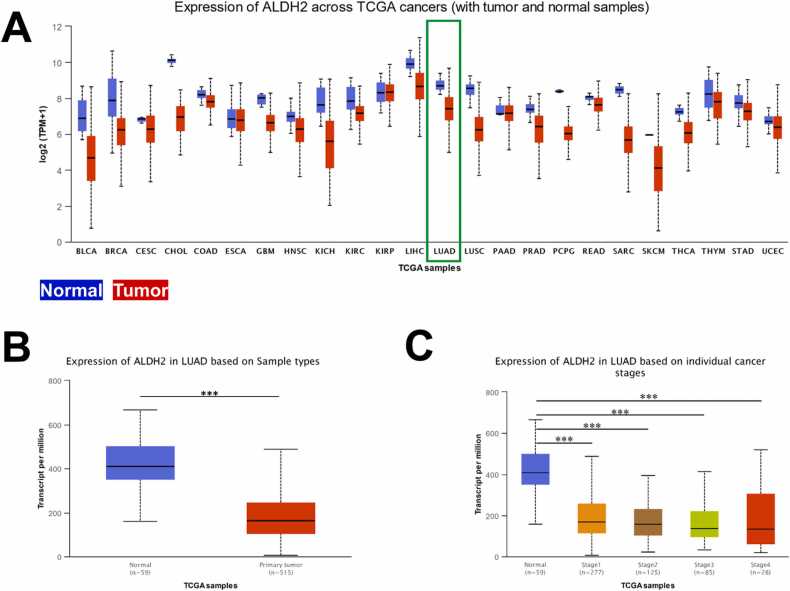
ALDH2 gene expression in lung adenocarcinoma. (A) UALCAN analysis of ALDH2 across 24 TCGA cancers including LUAD, tumor samples coded in red and normal samples coded in blue. The expression level of ALDH2 in LUAD based on (B) sample types and (C) on different LUAD stages, * **p < 0.001.

We evaluated ALDH2 gene expression across 24 different types of cancer including LUAD. Fig. 1A shows the downregulation of interested gene in 22 out of 24 investigated cancer types. With the aim to study the expression of ALDH2 in LUAD patients, we conducted the analysis of 59 normal and 515 primary tumor samples that collected from TCGA. The result revealed the mRNA expression levels of ALDH2 in LUAD tissues were significantly lower than in normal tissues, with a p-value of less than 1e-12 (Fig. 1B). In more detail, we took closer look into the expression of ALDH2 in different stages of LUAD patients. Fig. 1C demonstrates ALDH2 transcript in LUAD samples were significantly lower than normal samples at all four stages of LUAD (1st to 4th stage).

### 3.2 Weak ALDH2 protein expression in LUAD tissues

**Fig. 2:**
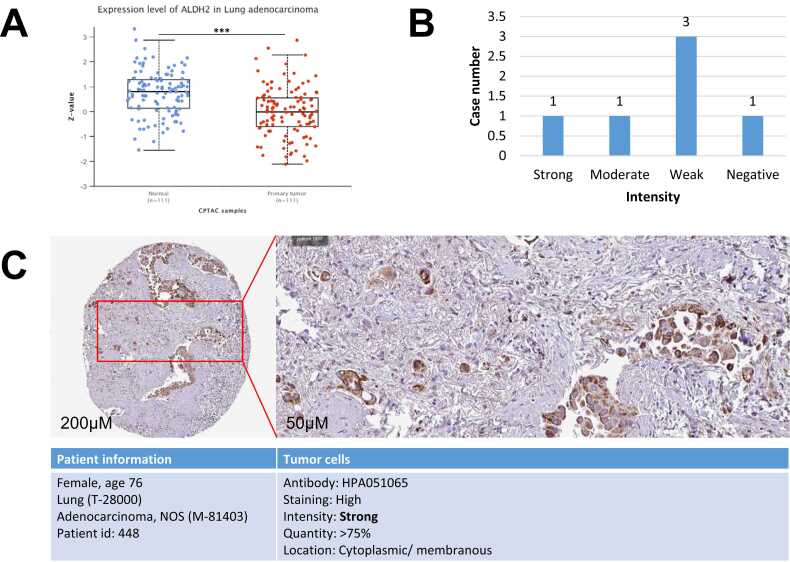
Protein expression of ALDH2 in LUAD samples. (A) UALCAN analysis 111 CPTAC cases illustrate the significant decreases of ALDH2 protein level in LUAD tumors. Z-values represent standard deviations from the median across samples, * **p < 0.001. (C) HPA provides the pathology of ALDH2 protein expression profiles and (B) statistical analysis. Data was collected on 25th January 2023.

The interested protein expression was investigated by using CPTAC-LUAD dataset and conducting via UALCAN software. The analysis of 111 normal and 111 LUAD samples show that ALHD2 protein expression is significantly lower in tumors compared to controls with p-value of 1.5e-08 (Fig. 2A). For visualization, the IHC images and protein profile of ALDH2 in LUAD tissues were collected from HPA. Fig. 2B depicts the weak expression of ALHD2 protein in.

LUAD tissues. In more detail, we found that there are three out of six staining tissues shows weak expression of ALDH2 in LUAD, one shows moderate expression, one shows strong expression and one sample shows the negative result. Fig. 2C illustrates the expression of ALDH2 in LUAD patient tissue stained by HPA051065 antibody, patient information was added.

### 3.3 ALDH2 has a strong prognostic value in LUAD patients

Li et al. reported in 2019 that ALDH2 repression is associated with a poor prognosis of LUAD [8]. However, the prognostic value of ALDH2 in combination with various clinicopathological factors is unknown. This study revealed the correlation between ALDH2 expression and clinical prognosis in LUAD patients with various clinicopathological factors. The KM Plotter analysis of 719 LUAD patients shows that low expression of ALDH2 remarkably impacts patient survival, with an HR of 0.48 and p-value of 8e-10. These results are expressed in Table 2. We found the relationship between ALDH2 expression, and LUAD patient survival time associated with various clinicopathological factors: cancer stage, smoking habit, gender, and treatment strategy.

**Table 2:** Analysis of the correlation between ALDH2 mRNA expression and clinical prognosis in LUAD patients with different clinicopathological factors by using Kaplan-Meier Plotter.

Lung Adenocacinoma	N	Hazard ratio	p-value
Overall survival	719	0.48 (0.37 − 0.61)	8e− 10
Clinicopathhological			
Stage			
1	370	0.37(0.24–0.57)	1.8e-6
2	136	0.73 (0.45 − 1.19)	0.2032
3	24	1.84 (0.67 − 5.08)	0.2307
AJCC stage T			
1	123	0.88 (0.48 − 1.62)	0.6878
2	105	0.36 (0.2 − 0.63)	0.0003
3, 4	Sample number too low for meaningful analysis		
AJCC stage N			
0	184	0.49 (0.3 − 0.8)	0.0034
1	44	0.52 (0.23 − 1.17)	0.1056
2	Sample number too low for meaningful analysis		
AJCC stage M			
0	231	0.47 (0.31 − 0.71)	0.0002
1	Sample number too low for meaningful analysis		
Gender			
Female	317	0.45 (0.3 − 0.67)	5.8e-5
Male	344	0.45 (0.32 − 0.63)	2.5e-6
Smoking history			
Include those never smoked	246	0.5 (0.31 − 0.81)	0.0042
Only those never smoked	143	0.35 (0.14 − 0.88)	0.0191
Surgery success			
Only surgical margins negative	204	0.41 (0.19 − 0.89)	0.0196
Chemotherapy			
No	21	1.53 (0.37 − 6.35)	0.5543
Yes	36	6.81 (1.64 − 28.32)	0.0035

Lower ALDH2 expression was significantly associated with a poorer OS in the first stage of LUAD, with an HR of 0.37 and a p-value of 1.8e-06. However, no relationship was identified between ADLH2 expression and survival in LUAD stage 2 and 3 patients. In addition, survival analysis based on the tumor-node-metastasis (TNM) system, one of the most widely used staging systems maintained by the American Joint Committee on Cancer (AJCC) and the International Union for Cancer Control (UICC), also was conducted. The stage grouping is classified based on T (the extent of the primary tumor), N (regional lymph nodes), and M (distant metastases). We discovered that LUAD patients with low ADLH2 expression had a worse OS in AJCC stage T2 with HR = 0.36 and p-value = 0.0003, whereas the patient number was insufficient for meaningful analysis in AJCC stages T3 and T4. Besides that, low ALDH2 expression patients with AJCC stage N0 had a worse overall survival (HR=0.49 and p-value = 0.0034). Regarding metastases, the low expression of ALDH2 in LUAD patients also correlated with poor OS at stage M0 (HR=0.47, p-value = 0.0002). These results suggested that we can use the low expression of ALDH2 to detect LUAD at a very early stage.

### 3.4 DNA methylation profile of ALDH2 in LUAD patients

**Fig. 3:**
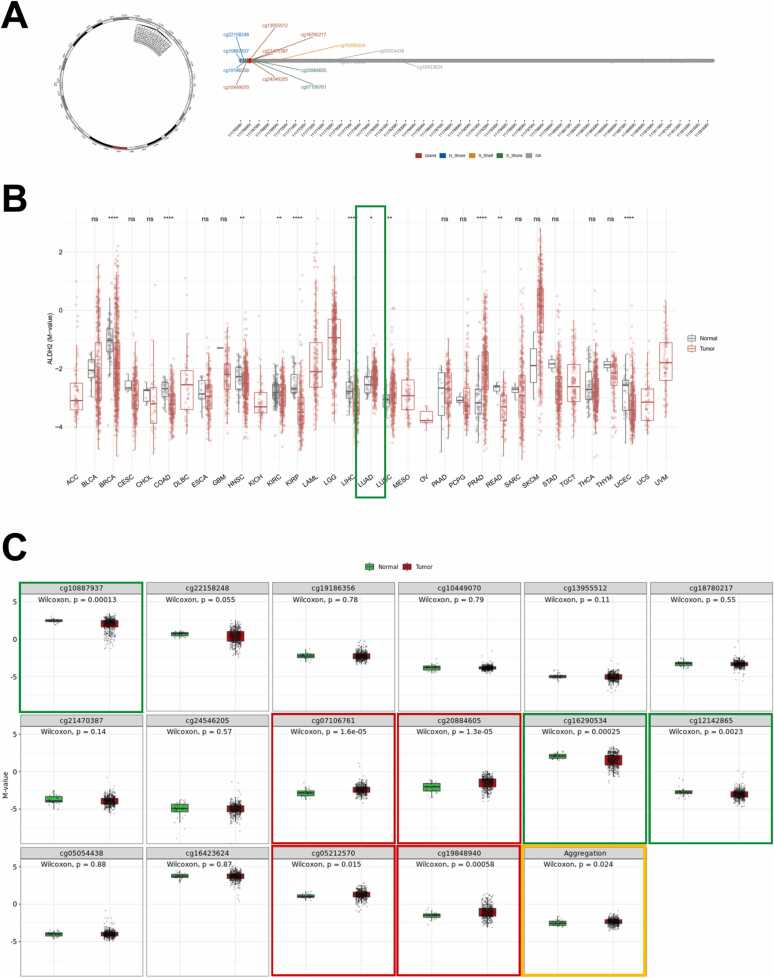
DNA methylation profile of ALDH2 in LUAD. (A) The chromosomal distribution of 16 methylation probes associated with ALDH2 on chromosome 12 and location of 16 probes in CpG Island. (B) ALDH2 methylation levels in tumor and normal samples cross different types of cancers. (C) Methylation of ALDH2 each probe and aggregation level. The significant higher methylated probes in LUAD compared to normal samples are bounded by a red box, the lower methylation level of LUAD samples is bounded by a green box, and methylation aggregation of ALDH2 in LUAD patients is bounded by an orange box. ns: p > 0.05; * : p < = 0.05; * *: p < = 0.01; * ** : p < = 0.001; * ** *: p < = 0.0001. Data was collected on 25th January 2023.

One of the most extensively researched epigenetic modifications in mammals is DNA methylation. It ensures proper gene expression regulation and stable gene silencing in normal cells [17]. In this study, we searched the DNA methylation level of ALDH2 in LUAD patients to figure out what was causing this gene's downregulation and low protein expression. Fig. 3A depicts chromosomal distribution of 16 ALDH2 methylation probes on chromosome 12 and demonstrates positions of 16 probes on CpG island (CGI). There are five probes are in Island region (cg13955512; cg18780217; cg21470387; cg24546205; cg10449070), three probes located in N_Shore region (cg10887937; cg22158248; cg19186356), two probes located in S_Shore region (cg07106761; cg20884605), one probe located in S_Shelf (cg16290534). The rest of five probes are non-identification.

We then investigated methylation levels of ALDH2 across 33 different cancer types including LUAD. We found that in LUAD samples, the ALDH2 methylation is higher than normal samples with p-value of 0.024. In other words, ALDH2 shows hyper-methylation status in LUAD patients (Fig. 3B and C). In more detail, we uncovered the methylation status of each probe (Fig. 3C). We found that there are three probes show significant hypo-methylation status (N_Shore_cg10887937; S_Shelf _cg16290534; non-identification_cg12142865) and four probes show significant hyper-methylation status (S_Shore_cg07106761; S_Shore_cg20884605; non-identification_cg05212570; non-identification_cg19848940).

### 3.5 ALDH2 get involved in a variety of signaling pathways

**Fig. 4:**
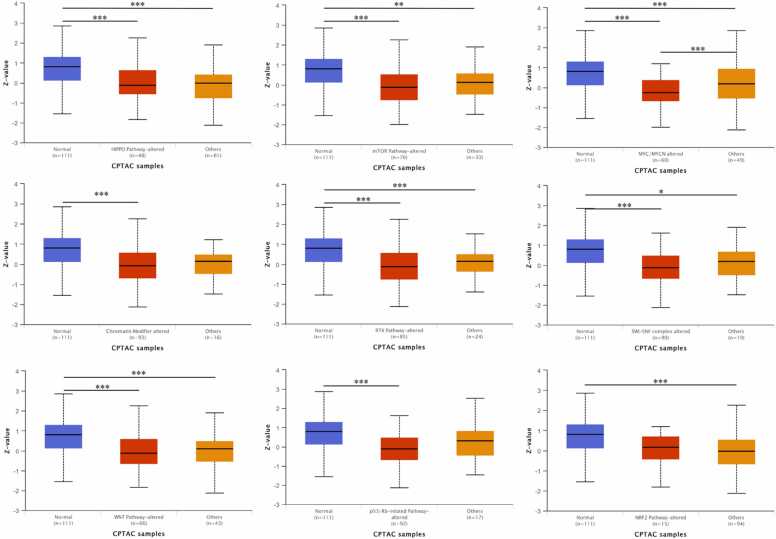
The ALDH2 proteomic expression profile based on different pathways in LUAD. UALCAN analysis total ALDH2 proteomic expression profile based on HIPPO, mTOR, MYC/MYCN, Chromatin modifier, RTK, SWI-SNF complex, WNT, p53/Rb and NRF2 pathway status. The CPTAC-LUAD including 111 cases was used. Protein expression of ALDH2 were quantified by mass spectrometry method. Z-values represent standard deviations from the median across samples for the given cancer type.

To search further into the role of ALDH2 in the LUAD pathogenic mechanism. We investigated potential ALDH2 signaling pathways involved in LUAD by using the CPTAC-LUAD dataset. The results showed strong correlations between ALDH2 proteomic expression and HIPPO Pathway, mTOR Pathway, MYC/MYCN Pathway, RTK Pathway, and SWI-SNF complex. Especially addition to the change of ALDH2 DNA methylation, ALDH2 proteomic expression level correlated to Chromatin Modifier alteration, another epigenetic modification. Noticeably, the protein expression level of ALDH2 was strongly related to the changes of two key stem cell-related pathways, including WNT and p53/Rb-related pathways. However, ALDH2 protein expression did not show correlation to the change in the inflammation-related NRF2 Pathway (Fig. 4).

### 3.6 The interaction of ALDH2 with stemness factors and immune system

**Fig. 5:**
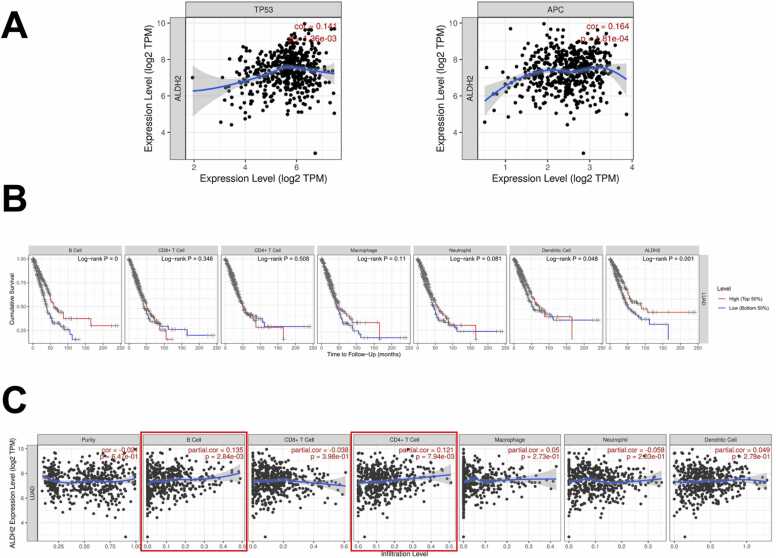
TIMER analysis the ALHD2 gene correlations and interactions of ALDH2 and immune cells in LUAD. (A) The correlation of TP53, APC and ALDH2. (B) The clinical relevance of LUAD to immune cells (B cell, CD8 +T cell, CD4 +T cell, Macrophage, Neutrophil, and Dendritic Cell) and ALHD2 expression, p-value of log-rank test for comparing survival curves of two groups is showed in each plot. (C) The correlation of ALDH2 with tumor purity and immune cells in the immune system shows the purity-corrected partial Spearman's rho value and statistical significance. Log2(TPM) is the log2 of the Transcript Count Per Million.

ALHD2 belongs to the ALDH superfamily, these family members were considered as stem cell markers in solid tumors [11]. Hence, in this study, we wanted to take closer look into the connection of ALDH2 with stemness-pathways. As above-noted, ALDH2 expression profile showed significant downregulation in two key stem cell-related pathways (WNT pathway and p53 pathway). Then, via TIMER analysis, we went further to investigate the correlation of interested gene with stemness factors including TP53 and APC (Adenomatous Polyposis Coli), the key WNT signaling pathway regulator. Fig. 5A illustrates the connection of ALDH2 with p53 (or TP53), correlation value of 0.14 (p = 0.00136) and with APC, correlation value of 0.164 (p = 1.81e-04).

The TIMER "Survival" study investigated the clinical relevance of ALDH2 expression and immune cell subsets in LUAD patients The results suggested that ALDH2 expression was significantly related to LUAD patient survival time (log-rank p of 0.001). Regarding different immune cells, B cells and dendritic cells show a significant correlation to the survival time of LUAD patients with the p-value of 0.0003 and 0.048, respectively (Fig. 5B).

We then looked into the relationship between ALDH2 and various immune cell activities. Fig. 5C shows that ALDH2 has the highest association with B cell activity (partial correlation of 0.135, p = 0.00284). And ALDH2 had a partial correlation with CD4 +T cell activity (correlation value of 0.121, p = 0.00794). These findings suggest that ALDH2 interacts with B and T cells in LUAD patients.

## 4 Discussion

### 4.1 ALDH2 as a biomarker for early detection of LUAD

Early detection of cancer, particularly LUAD, usually increases the likelihood of successful treatment for patients. LUAD mortality and incidence remain high, despite advances in treatment and diagnosis. Most patients are diagnosed in the late stage because early symptoms are not obvious. Hence, to improve the prognosis of LUAD patients, we attempted to identify potential biomarkers for LUAD early screening by using bioinformatics analysis.

Previous research has demonstrated the role of ALDH2 in variety of diseases. ALDH2 has been shown as an essential enzyme in protecting the heart against oxidative stress via the depletion of 4-Hydroxynonenal (4-HNE) [18]. The metabolism of reactive oxygen species (ROS) and 4-HNE is deeply involved in cancer cell death. Low ALDH2 expression leads to the accumulation of aldehydic products such as acetaldehyde, 4-HNE, and malondialdehyde (MDA), all of which are associated with high cancer morbidity [19], [20]. In current study, we found significant downregulation of ALDH2 transcriptomic and protein levels in LUAD patients, which was consistent with the finding from Yang et al. [9]. In addition, the reduced expression of ALDH2 in LUAD tissues compared to normal tissues suggested us to further look at the related DNA methylation mechanism. We observed hyper-methylation status of ALDH2 in LUAD with four evident hyper-methylated probes and three hypo-methylation probes. This explores add to the knowledge from Yang et al.’s study that DNA methylation of the ALDH2 gene locus leads to loss of expression of ALDH2 [21]. Furthermore, we uncovered ALDH2 expression correlates to the survival time of LUAD patients. At this point, our result is consistent to Chen’s study in 2018 and the Li’s study in 2019. Chen et al. reported that ALDH2 expression was found to be negatively correlated with DNA base excision repair protein (XRCC1) expression, suggesting that low ALDH2 expression is responsible for poor overall survival in lung and liver cancer patients [22]. Li et al. reported that poor prognosis in LUAD was associated with ALDH2 repression [8]. Here, more importantly, we discovered the significant association between ALDH2 expression and OS of LUAD in the early stage.

Our findings indicate that ALDH2 is downregulated in LUAD patients, has aberrant DNA methylation, and is related to LUAD patient survival time. These findings strongly suggest ALDH2 as a potential marker for the prognosis of early-stage LUAD.

### 4.2 ALDH2 could be a stem cell-related biomarker for targeting LUAD treatment

Gene-targeting, immunomodulatory, and cell-based treatments will be potentially useful strategies for lung cancer treatment in the era of personalized medicine [23]. Despite substantial breakthroughs in target treatment and immunotherapy, LUAD patient survival remained stagnant, particularly in late stages [24]. Hence, it is an urgent issue to figure out a stem cell-related gene that could be a target for novel therapeutic strategies.

The research on the role of ALDH2 in CSC hallmarks is limited and contentious. In 2019 Li et al. found that treating LUAD cells with the ALDH2 agonist Alda-1 inhibited their proliferation, stemness, and migration [8]. However, according to Chen et al. (2020), silencing FOXM1 inhibits stemness of liver cancer stem cells (LCSCs) by decreasing ALDH2 expression, and represses LCSC proliferation, migration, invasion, and tumorigenesis while inducing apoptosis [25]. It is evident that ALDH2 expression in CSCs hallmarks appears complicated and ambiguous. Further study is needed to clarify the mechanism of ALDH2 in stem cell-related pathways.

In this study, our results support the idea of ALDH2 downregulation is correlated to stem cell-related pathways including WNT and p53. We demonstrated the significant downregulation of ALDH2 proteomic in WNT and p53 pathways and reported the correlation of ALDH2 with p53 and APC, the key WNT regulator. The Wnt/beta-catenin signaling system regulates the delicate balance of stemness and differentiation in various adult stem cell niches, including cutaneous hair follicles, the mammary gland, and the intestinal crypt [26]. As a result, constitutive WNT signaling activation induced by mutations in genes encoding its downstream components drives cancer in these organs. The rate-limiting event in most sporadic colorectal cancer cases is either loss of APC function or oncogenic beta-catenin mutations [26]. Aside from its established roles, emerging data show that p53 is vital in controlling stem cell homeostasis [27]. p53 suppression or deletion boosts reprogramming efficiency by 3–10-fold in investigations involving the reprogramming of differentiated cells into induced pluripotent stem cells [28], [29], [30]. Hence, our findings suggest that ALDH2 downregulation in LUAD may have occurred via WNT and p53 stem cell-related pathways. However, more research is needed to determine whether ALDH2 is a lung cancer stem biomarker that can be used to target LUAD treatment.

## 5 Conclusion

This study demonstrated the clinical significance of ALDH2 for early diagnosis of LUAD. Additionally, we indicated the hyper-methylation status of ALDH2 in LUAD patients. Also, we uncovered the mechanism of ALDH2 associated with the stem cell-related pathways; this suggested a potential target for treatments. We also reported the correlation of ALDH2 with the activities of immunological B cells and CD4 +T cells. Taken together, we provided substantial evidence to support the clinical application of ALDH2 as a prognosis biomarker in early-stage of LUAD. Further analysis regarding the stem cell-related pathways of ALDH2 for LUAD treatment should be investigated in more depth.

## Ethics approval and consent to participate

Ethical review and approval were waived for this study because all data were derived from the public databases.

## Funding

This work was supported by the National Science and Technology Council, Taiwan [grant numbers MOST110-2221-E-038-001-MY2 and MOST111-2628-E-038–002-MY3].

## Consent for publication

Consent for publication was waived for this study because all data were derived from the public databases.

## CRediT authorship contribution statement

**Thi-Oanh Tran:** Conceptualization, Methodology, Formal analysis, Investigation, Writing – original draft, Writing – review & editing, Visualization. **Thanh Hoa Vo:** Methodology, Formal analysis, Writing – original draft, Writing – review & editing. **Luu Ho Thanh Lam:** Formal analysis, Writing – review & editing. **Nguyen Quoc Khanh Le:** Conceptualization, Methodology, Validation, Writing – review & editing, Visualization, Supervision, Funding acquisition.

## Competing interests

The authors declare no conflict of interest.

## Data Availability

All data were derived from the public databases (TCGA and CPTAC).
